# Zebrafishing for enhancers of hearing regeneration

**DOI:** 10.1016/j.xgen.2022.100178

**Published:** 2022-09-14

**Authors:** Karen Echeverri

**Affiliations:** 1 Eugene Bell Center for Regenerative Biology and Tissue Engineering, Marine Biological Laboratory, Woods Hole, MA, USA

## Abstract

The discovery of regeneration-specific enhancer elements has added an exciting player to the field of regeneration biology. In this issue of *Cell Genomics*, Jimenez et al.^1^ demonstrate the power of combining single-cell genomics with the genetically tractable zebrafish to identify modulators of adult hair cell regeneration.

## Main text

Hearing loss affects people of all ages, but the incidences increase significantly with age. Loss of hearing is due to infection, loud noise, traumatic injury, and age. Specialized hair cells located deep in the inner ear convert sounds into nerve impulses that are transmitted to the brain; these cells are irreplaceable in humans after damage. Although humans can regenerate some cell types, we cannot regenerate hair cells of the ear.

An increasing number of organisms have been identified that possess inherent regenerative abilities, with a number exhibiting an ability to regenerate hair cells. A variety of vertebrate animals, including salamanders, sharks, bony fish, rays, amphibians, reptiles, and birds, can replace hair cells throughout their life. Cell-tracing studies, especially in the lateral line organ of salamanders and zebrafish, confirm that the support cells, a lineage-restricted hair stem cell population, give rise to the new hair cells.^2^

In humans, the inner ear consists of the cochlea, responsible for hearing, and the semicircular canals, which regulate balance. Humans have support cells in the inner ear along with hair cells, but the damaged hair cells cannot be replaced in adults, suggesting a genetic program restricting them from responding to injury. Thus, there is much interest in understanding the cellular and molecular mechanism driving this phenomenon.

The zebrafish, *Danio rerio*, has featured in studies of the development and regeneration of hair cells, including comparative studies across species. Fish ears are not easily accessible for techniques like *in vivo* imaging. However, zebrafish have a lateral line organ located at regular intervals along the surface of the body. In the lateral line, the hair cells are clustered into structures termed neuromasts and are used to sense the water and maintain balance.^3^ Because of their accessibility, the lateral line is a popular system in which to study hair cell regeneration. Like human hair cells, these zebrafish hair cells are damaged by exposure to antibiotics, loud noise, and drugs.^4^^,^^5^ Amazingly, within 48 h, zebrafish hair cells can regenerate with both form and function restored after hair cell death. In this model, the key genes driving the cycle of homeostatic replacement of hair cells and the regeneration of damaged hair cells have been identified.^4^ Nevertheless, the degree of similarity of zebrafish hair cells to human ears is still unknown, as is why human ear support cells do not respond similarly to injury.

**Figure 1:**
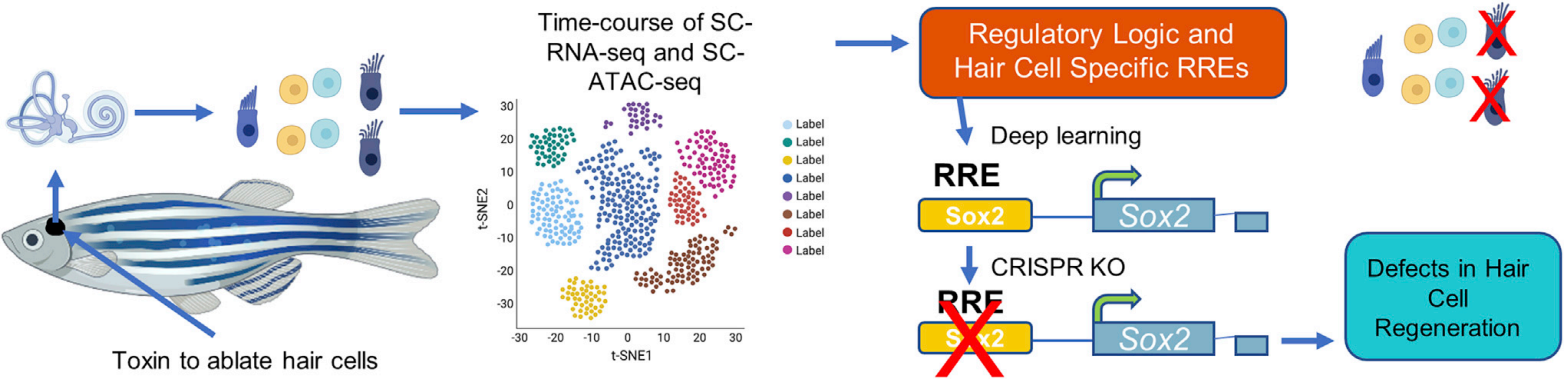
Identifying enhancers of adult hair cell regeneration Jimenez et al.^1^ use the power of the genetically tractable zebrafish to ablate adult hair cells, isolate single cells, and carry out single-cell sequencing and single-cell ATAC sequencing. This approach enabled identification of hair-cell-specific regeneration enhancers. They then focused in on a regeneration-specific enhancer element of the Sox2 gene, which could be genetically deleted using CRISPR, leading to defects in hair cell regeneration

Jimenez et al.^1^ exploited the genetic tractability of the adult zebrafish by utilizing a transgene expressed only in adult hair cells linked to a diphtheria toxin receptor in a manner that specifically ablates mature hair cells in the saccule and utricle, without affecting the support cells. This allowed them to examine the response of cells in the organ over time. They followed hair cell apoptosis (day 4), hair cell death, the initiation of the regeneration program (day 5), and when hair cells begin to repopulate the sensory epithelium (day 7). A single-cell epigenome and transcriptome were generated and compared to cells isolated from adult uninjured ear cells. This allowed the authors to assign cell-type identities to clusters of cells with expression patterns from previous transcriptomes of the zebrafish inner ear and lateral line.^6^, ^7^, ^8^ This elucidated key differences between the cells of the inner ear and the lateral line organ. This study contains a wealth of information that can be mined to identify cell markers for both inner ear and lateral line organs and the molecular mechanisms driving activation versus termination of regeneration (Figure 1).

Uniquely, Jimenez et al.^1^ identify regeneration-specific responses of support cells in the inner ear over the course of the regeneration process. Single cell ATAC-seq provides data on chromatin accessibility over the time course of injury to regeneration. Regions of chromatin displaying higher accessibility during regeneration are termed “regeneration-responsive elements” (RREs). Many RREs were identified in each cell population, with the highest overlap found between the progenitor cells and hair cells, while the support cells appeared more distinct. Deep-learning algorithms helped distinguish a cell-type-specific enrichment of Six and Sox motifs, with Sox motifs more enriched in support cells. Both genes are expressed in developing hair cells, and knockouts in these gene families result in ear development defects. The authors identify a switch-like pattern in sox2 expression during regeneration. Deleting a 2 kb enhancer element of sox2 resulted in no morphological defects in larval or adult stages; however, both homozygous and heterozygous larval animals failed to regenerate normal numbers of neuromasts in their lateral line after treatment with copper sulfate. Additionally, transgenic fish carrying cell-specific diphtheria toxin also failed to regenerate normal numbers of hair cells. Interestingly, deleting the enhancer shifts the timing of sox2 expression, suggesting that coordinated timing of sox and six genes may regulate the number of new hair cells regenerated. These data suggest that a regeneration-specific enhancer of the sox2 gene controls the timing of sox2 gene expression during regeneration. It is unclear whether sox2 is necessary to regulate the support or progenitor pool number or whether it controls the switch from proliferation to differentiation. Importantly, this study identifies and functionally tests the first adult regeneration-specific enhancer in the fish ear.

Previous work has identified injury-responsive enhancers in the zebrafish heart; however, these were not essential for driving the regeneration response.^9^ Recently fish RREs were identified that drive a regeneration response program in the caudal fin, suggesting separate injury and regeneration programs.^10^ It may be that mammals experienced selection for the injury response program and lost the regeneration-specific program, resulting in wound healing and scarring instead of a pro-regenerative program. New technological advances, like rapid sequencing of new genomes and single-cell ATAC sequencing will allow us to look more broadly across regeneration-competent species to understand how enhancers may have been repurposed, diverged, or were lost and to take advantage of genetically tractable organisms to functionally test these findings *in vivo*, working toward the ultimate long-term goal of enhancing regeneration in the hearing impaired.
